# Contribution of genetic effects to genetic variance components with epistasis and linkage disequilibrium

**DOI:** 10.1186/1471-2156-10-52

**Published:** 2009-09-04

**Authors:** Tao Wang, Zhao-Bang Zeng

**Affiliations:** 1 Division of Biostatistics, Department of Population Health, Medical College of Wisconsin, Milwaukee, WI 53226, USA; 2 Bioinformatics Research Center, Department of Statistics, North Carolina State University, Raleigh, NC 27695, USA

## Abstract

**Background:**

Cockerham genetic models are commonly used in quantitative trait loci (QTL) analysis with a special feature of partitioning genotypic variances into various genetic variance components, while the F_∞ _genetic models are widely used in genetic association studies. Over years, there have been some confusion about the relationship between these two type of models. A link between the additive, dominance and epistatic effects in an F_∞ _model and the additive, dominance and epistatic variance components in a Cockerham model has not been well established, especially when there are multiple QTL in presence of epistasis and linkage disequilibrium (LD).

**Results:**

In this paper, we further explore the differences and links between the F_∞ _and Cockerham models. First, we show that the Cockerham type models are allelic based models with a special modification to correct a confounding problem. Several important moment functions, which are useful for partition of variance components in Cockerham models, are also derived. Next, we discuss properties of the F_∞ _models in partition of genotypic variances. Its difference from that of the Cockerham models is addressed. Finally, for a two-locus biallelic QTL model with epistasis and LD between the loci, we present detailed formulas for calculation of the genetic variance components in terms of the additive, dominant and epistatic effects in an F_∞ _model. A new way of linking the Cockerham and F_∞ _model parameters through their coding variables of genotypes is also proposed, which is especially useful when reduced F_∞ _models are applied.

**Conclusion:**

The Cockerham type models are allele-based models with a focus on partition of genotypic variances into various genetic variance components, which are contributed by allelic effects and their interactions. By contrast, the F_∞ _regression models are genotype-based models focusing on modeling and testing of within-locus genotypic effects and locus-by-locus genotypic interactions. When there is no need to distinguish the paternal and maternal allelic effects, these two types of models are transferable. Transformation between an F_∞ _model's parameters and its corresponding Cockerham model's parameters can be established through a relationship between their coding variables of genotypes. Genetic variance components in terms of the additive, dominance and epistatic genetic effects in an F_∞ _model can then be calculated by translating formulas derived for the Cockerham models.

## Background

Genetic models provide a basis for analyzing genetic properties in study populations. For quantitative traits, one type of models that has long been used in experimental designed populations for analysis of quantitative trait loci (QTL) is the so-called Fisherian or Cockerham model with a focus on partition of genotypic variances into additive, dominance and epistatic genetic variance components, and their model parameters are often called average allelic effects. Another popular model which has been widely used in many genetic association studies is referred to as the F_∞ _model whose parameters are often defined as the additive, dominance and epistatic effects [1-4]. Over years, there have been some confusion about the relationship between these two types of models [5-7]. The relationship between the additive, dominance and epistatic effects and the genetic additive, dominance and epistatic variance components has not been well established, especially when multiple QTL are involved in presence of epistasis and linkage disequilibrium (LD). To shed some light on this issue, in this paper we further explore the differences and links between these two types of models.

In genetic studies, a partition of genotypic variance into additive, dominance and epistatic variance components provides us a basis to better understand the genetic inheritance properties of a quantitative trait from a parental population to their progeny population. For example, the additive variance is the chief cause of resemblance between relatives. The genetic variance components also play a crucial role in studies of heritability, covariance between relatives, and variance components analysis. Fisher [8] proposed a least square regression model for modeling QTL by partitioning genotypic variance into additive, and dominance variance components, where an additive variance describes the variation contributed by an average substitution effect of a specific allele transmitted from a parent to offspring, and where a dominance variance is a portion of the genotypic variance due to interaction of the two alleles from both parents. Cockerham [9,10] extended the Fisher's model to multiple loci with redefined additive, dominance and epistatic effects of QTL based on statistical orthogonal contrasts. Kempthrone [11,12] further generalized the model to multiple alleles. More recently, Mao *et al*. [13] extended the two-locus biallelic Cockerham model to allow Hardy-Weinberg and linkage disequilibria, and they introduced 35 allelic effect parameters to describe various allelic effects and their interactions. Zeng *et al*. [7] introduced a general multi-locus-two-allele (G2A) model to represent the Cockerham model in a multiple regression model setting, and compared several models for analyzing QTL effects and epistasis. Wang and Zeng [14] further extended the approach to multiple alleles and derived formulas for computing variance components in presence of epistasis and LD.

The F_∞ _model focuses on direct modeling of genotypic values and testing for genotypic association of QTL with quantitative traits. There have been discussions on classification of various penetrance modes of diseases based on the F_∞ _model and genotypic values [15]. In terms of its modeling scheme, as we will see later in this paper, the F_∞ _model is genotype-based by treating genotypes as different levels of the locus factors. Álvarez-Castro and Carlborg [16] also proposed a unified model to incorporate both genotypic and allelic effects into one framework. Meanwhile, there have been continuous efforts on modeling QTL effects and epistasis based on their biological functions [5,6,17]. More recently, several articles have addressed the issue of F_∞ _models on partition of genotypic variances [7,18,19]. Under the assumption of linkage equilibrium, Tiwari and Elston [19] considered a two-locus biallelic F_∞ _model and derived formulas for computing genetic variance components in terms of the additive, dominant and epistatic genetic effects. Yang [18] discussed the impact of zygotic association on partition of genotypic variance in F_∞ _models. Zeng *et al*. [7] compared the difference in definition of model parameters between the F_∞ _and Cockerham models. It was pointed out that these two types of models are different ways of modeling the genotypic values and the two models' parameters are transferable from one to the other through their relationship with the genotypic values under certain circumstances.

In this paper, we further explore the differences and links between the F_∞ _and Cockerham models in terms of their modeling schemes and on partition of the genotypic variance. First, we clarify that the Cockerham type models are in fact allele-based models with a special modification to correct a collinearity problem. Formulas of several moment functions for a two-locus biallelic Cockerham model are also derived, which are useful for deriving formulas in calculation of the genetic variance components. Next, we explore the difference and properties of these two types of models in partition of genotypic variances. We show that the traditional F_∞ _models are basically genotype-based models in which the additive and dominance effects could be confounded with each other in partition of genotypic variances. This fact usually does not affect association tests in the standard regression analysis. But it can make the partition of genotypic variances intricate, especially when locus-by-locus interactions are involved. We also introduce a mean-corrected F_∞ _model, which can provide a partially orthogonal partition of the genotypic variance between loci under zygotic equilibria, although its within-locus variances may still not be orthogonal due to the possible confounding between its coding variables of genotypes. We discuss pros and cons of the F_∞ _and Cockerham models in association analysis and in partition of the genotypic variances.

The second part of this paper concentrates on calculation of the genetic variance components in terms of the additive, dominance and epistatic genetic effects in an F_∞ _model. Tiwari and Elston [19] derived formulas for computing genetic variance components in terms of the additive, dominant and epistatic genetic effects for a two-locus biallelic F_∞ _model under the assumption of linkage equilibrium. More recently, Zeng *et al*. [7] proposed a way of linking the two sets of model parameters through their relationship with the genotypic values when fully parameterized models are applied. Since formulas for partition of the genotypic variance into additive, dominance and epistatic variances have been well established for Cockerham models [9,14], we can then calculate the genetic variance components by translating the partition formulas of the variance components derived from their equivalent Cockerham models. As examples, for a one-locus F_∞ _model with Hardy-Weinberg disequilibrium and a two-locus F_∞ _model with both epistasis and LD, we present detailed formulas for computing various genetic variance components in terms of the additive, dominant and epistatic effects together with allele frequencies and LD measures. We also propose an alternative way of transforming the additive, dominance and epistatic effects in an F_∞ _model into the average allelic effects in its corresponding Cockerham model through the coding variables of genotypes used in these two models, which is especially useful when reduced F_∞ _models are applied. Moreover, Some practical issues relating to using of reduced F_∞ _or Cockerham models are addressed.

## Results

### Genetic models

In the analysis of quantitative trait, the observed phenotypes can usually be expressed through the following model

(1)

where Y is the phenotypic value, G is the genotypic value, E is the environmental deviation, and *G *× *E *is the genetic by environmental interaction. Adjustment for environmental deviation and genetic by environmental interaction can usually be achieved by incorporating suitable environmental covariates into the model. Therefore, in the rest of the paper, we omit *E *and *G *× *E *from the model and focus on modeling and analysis of the genotypic values.

Quantitative trait loci (QTL) refer to genes that contribute to variation of a quantitative trait. In a study population, given specific genotypes *g *at the QTL under consideration, the genotypic value *G*(*g*) = *E*(*G*|*g*) is defined as the mean of individuals with genotypes *g *in the study population. In practice, the genotypic value *G *of an individual is unknown and needs to be estimated. Let *P*_*g *_be the genotypic distribution of the QTL in the study population, a regression model can be expressed as



where the genotypic value *G*(*g*) is fixed given a specific genotype *g*. Since the QTL usually has a finite number of genotypes, *G*(*g*) itself can be treated as a discrete random variable that takes certain quantitative values with its distribution specified by *P*_*g*_. Therefore,



With a large enough random sample from a study population, the genotype data from the sample would follow approximately the same genotypic distribution as *P*_*G*_. The classical analysis of variance (ANOVA) or regression analysis is a typical tool for analysis of *V*_*G *_and test for possible association of genotypes at the QTL with the phenotypic trait. Now, a fundamental question is how to model the genotypic values G(g) given the QTL genotypes.

In human genome, an individual always carries two alleles at a QTL - one from the father and the other from the mother. It is possible that a disease is caused by a mutant allele inherited from one of the parents. To understand such inheritance properties from parents to their offspring, a natural way is to treat paternal and maternal alleles as two different factors and assess their allelic effects. Given that, let us first consider a single QTL case with two alleles *A*, *a *at the locus. For each individual, we can define the following indicator variables to describe the transmission of alleles from parents to the individual.



Then we can write down a simple regression model as



where *g *= (*a*, *a'*) with *a*, *a' *being the paternal and maternal allele, respectively. In practice, however, this model is not very useful because we usually cannot distinguish the paternal and maternal alleles from the observed genotype data; i.e., the so-called phase problem. But suppose that the paternal and maternal alleles have the same effects, which is a reasonable assumption in most of the genetic studies, then the above model can be simplified as

(2)

where *w'*(*g*), *v'*(*g*) are defined as



In this model, based on the genotypic values, we have *α' *= *G*_*Aa *_- *G*_*aa*_, *δ' *= (*G*_*AA *_+ *G*_*aa*_) - 2*G*_*Aa*_, and the reference point (or baseline) *μ' *= *G*_*aa *_is the genotypic value of genotype *aa*.

Typically, the genetic additive variance *V*_*A *_is defined as a variation contributed by allelic effects alone, and the genetic dominance variance *V*_*D *_is the variation contributed by interaction of the paternal and maternal alleles. Under the assumption of Hardy-Weinberg equilibrium (HWE), it is well known that the genotypic variance has an orthogonal partition *V*_*G *_= *V*_*A *_+ *V*_*D *_in which the genetic dominance variance *V*_*D *_becomes the deviation of the genetic variance attributable to the locus from the additive variance [4,20]. A first look at model (2) might lead us to believe that under HWE we would have an orthogonal partition of the genotypic variance *V*_*G *_= *V*_*A *_+ *V*_*D *_with *V*_*A *_= *V *(*α'w'*(*g*)) and *V*_*D *_= *V *(*δ'v'*(*g*)). However, this is not true because the interaction term *δ'v'*(*g*) in model (2) is correlated with the additive term *α'w'*(*g*) due to a positive correlation between *z*_*M *_(or *z*_*F*_) and *v' *= *z*_*M *_*z*_*F*_. In fact, although the two indicator variables *z*_*M *_and *z*_*F *_are assumed to be independent under HWE, we have covariances Cov(*z*_*M*_, *z*_*M *_*z*_*F*_) = Cov(*z*_*F*_, *z*_*M *_*z*_*F*_) = *V *(*z*_*F*_)*E*(*z*_*M*_) = *p*^2^(1 - *p*), where *p *= *p*_*A *_is the frequency of allele *A*. Therefore, the covariance between the two coding variables *w' *and *v' *is Cov(*w'*, *v'*) = Cov(*z*_*M *_+ *z*_*F*_, *z*_*M *_*z*_*F*_) = 2*p*^2^(1 - *p*), which means *w' *and *v' *are almost always positively correlated as long as the frequency of allele *A *not being zero. Even more general, from the definition of *w' *and *v' *above, we can show that Cov(*w', v'*) = 2(1 - *p*)*P*_*AA*_, regardless of whether there is HWE or not. Thus, model (2) provides a partition of the genotypic variance as



with a portion of it contributed by correlation between the effects *α' *and *δ'*. This problem, caused by using two correlated explanatory variables *w'*, *v' *in a multiple regression model, is often referred to as a confounding problem, or statistically, a multicollinearity problem, which tends to make and partition of variance components and the interpretation of the regression coefficients intricate, and in extreme cases leads to large standard errors for the least square estimates. To overcome this multicollinearity problem on partition of genetic variances, one strategy is to make mean corrections on those genotype coding variables [7,14]. If we introduce two mean-corrected index variables defined by *x*_*M *_= *z*_*M *_- *p *and *x*_*F *_= *z*_*F *_- *p*, then we can build a modified version of model (2) as in the following

(3)

where *w*(*g*), *v*(*g*) are defined by



It should be pointed out that the index variable *v *as defined above is slightly different by (-2) folds from the one we defined in [14] in order to keep the definition of *δ *consistent with the G2A model introduced in Zeng *et al*. [7], of which the standard F_2 _model is a special case.

Model (3) is actually a regression form of the Cockerham model in one QTL case [7]. Under HWE, the indicator variables *z*_*M *_and *z*_*F *_are independent, as well as the index variables *x*_*M *_and *x*_*F*_. Thus we have now , which leads to our familiar orthogonal partition of the genotypic variance *V*_*G *_= *V*_*A *_+ *V*_*D *_with *V*_*A *_= *α*^2^*V *(*w*) = 2*α*^2^*pq *and *V*_*D *_= *δ*^2^*V *(*v*) = 4*δ*^2^*p*^2^*q*^2^, where *q *= 1 - *p*. Under Hardy-Weinberg disequilibrium, we can represent genotype frequencies as *P*_*AA *_= *p*^2 ^+ *pqf*, *P*_*Aa *_= 2*pq *- 2*pqf *and *P*_*aa *_= *q*^2 ^+ *pqf*, where f is a measure of departure from HWE. Then the genotypic variance *V*_*G *_= *V*_*A *_+ *V*_*D *_+ 2Cov(*A*, *D*) with



Back to the previous model (2), it is easy to see that the coding variables *w'*, *v' *in model (2) and the index variables *w*, *v *in model (3) have relationships *w' *= *w *+ 2*p *and . Note that *w' *is still the one that specifies the additive effect except with a constant shift, whereas *v' *includes a portion of *w*, which is the reason why model (2) cannot provide orthogonal partition of genotypic variance under HWE. The positive correlation between the two coding variables *w' *and *v' *in model (2) can also complicates the interpretation of regression parameters *α'*, *δ'*. Using the method proposed in the next section, we can show that the parameters in models (2) and (3) have relationships *α' *= *α *+ 2*pδ *and *δ' *= -2*δ*. Thus, the additive effect *α' *in model (2) is actually a combination of the average allelic effect *α *and dominance effect *δ *in the Cockerham model (3). On model (2) in partition of genotypic variance, we have under HWE



where *V *(*w*) = 2*pq*. Note that *V*_*A *_= *α*^2^*V *(*w*) = 2*pqα *^2^. So, the positive correlation between the two coding variables *w' *and *v' *leads to an increased share of *V *(*δ'v'*) other than *V*_*D*_, which is partly contributed by a portion of the additive variance. By using the mean-corrected index variables *w *and *v*, the Cockerham model allows us to separate the confounding effects of the two variables *w' *and *v' *at least under HWE in partition of genotypic variance *V*_*G*_. As a result, the dominance variance *V*_*D *_in the Cockerham model (3) is the *additional *variation contributed by interaction of the paternal and maternal alleles, in addition to the additive variance.

The Cockerham model (3) can easily be extended to multiple loci. For example, consider two loci **A **and **B **with alleles *A*, *a *and *B*, *b*, respectively. We can define indicator variables:



and



for the two loci separately. By further introducing  and , where *p*_1 _= *P*_*A*_, *p*_2 _= *P*_*B*_, and assuming that paternal and maternal gametes (alleles and haplotypes) have the same genetic frequencies and effects, we obtain the following two-locus (G2A) Cockerham model [14]

(4)

where



Based on these mean-corrected index variables, this Cockerham model allows us to easily incorporate some allelic related properties, such as HWE or linkage equilibrium information, into the variance partition analysis [14]. For instance, since the means of the *x'*s variables are scaled to zero in the population, it is easy to see that all the components in model (4) are independent with each other under Hardy-Weinberg and linkage equilibria, which leads to orthogonal partition of variance components. In addition, those mean-corrected variables *x'*s defined above have some nice properties that can facilitate derivation of formulas for various variance and covariance components. For example, for two loci **A **and **B **under HWE but with LD between them, we can show through some derivation that for any integers *m*, *n *> 0



where *q*_1 _= 1 - *p*_1_, *q*_2 _= 1 - *p*_2 _and *D *= *P*_*AB *_- *p*_1 _*p*_2_. These moment functions are quite useful in deriving formulas for partition of the genotypic variance into various allelic based variance components for the above G2A Cockerham model. Besides, under gametic equilibrium, ,  are independent of , . Hence, for any *j*, *k *= 1, 2 and integers *m*, *n *> 0. Moreover,  and , as we do not distinguish the paternal and maternal gametes.

Note that the above model (4) uses 9 parameters to model G(g), which contains 9 parameters. So this is also a fully parameterized model. In other words, the model parameters *E*_*G*2*A*·*AB *_= (*μ*, *α*_1_, *δ*_1_, *α*_2_, *δ*_2_, (*αα*), (*αδ*), (*δα*), (*δδ*))^*T *^simply provide a re-parameterization of the 9 genotypic values *G*_*AB *_= (*G*_22_, *G*_21_, *G*_20_, *G*_12_, *G*_11_, *G*_10_, *G*_02_, *G*_01_, *G*_00_)^*T*^, where *G*_*ij*_, *i*, *j *= 0, 1, 2, denote genotypic values with *i*, *j *being the counts of *A*, *B *alleles in the corresponding genotypes. Using the same notation as in Zeng *et al*. [7], we have  where



As pointed out in [7], the above relationship holds regardless of whether there is a linkage equilibrium or disequilibrium in the study population.

In genetic association studies, we are often interested in examining association of genotypes at certain genetic markers or QTLs with a disease phenotype. In this case, a standard approach is to fit a regression model with genotypes at each locus being treated as different levels of the locus factor. This leads to another popular type of models that have been widely used in genetic association studies; i.e., the so-called F_∞ _models. Still, let us first consider the simple case of one locus with two alleles *A*, *a*. In this case, we have three possible genotypes *AA*, *Aa *and *aa*, and correspondingly three possible genotypic values *G*_*AA*_, *G*_*Aa *_and *G*_*aa*_. The single locus F_∞ _model is then given by [3,4]



where *a*, *d *are often called the additive, dominance effects of alleles *A*, *a*, respectively. In terms of the genotypic values, the additive and dominance effects are defined as , , . This model is referred to as an F_∞ _model simply because the reference point *m *in the model is the mean of two homozygote genotypic values which corresponds to the mean in an F_∞ _population [1,2].

The above model can also be written in a regression model form as

(5)

where w* (*g*), v* (*g*) are two coding functions of genotypes *g *which are defined as



Since *m*, *a *and *d *in this model simply provides a re-parameterization of the original three genotypic values *G*_*AA*_, *G*_*Aa *_and *G*_*aa*_, we can refer *a*, *d *as genotypic effects of the QTL with *m *as a reference baseline.

Statistically, in order to see whether the QTL genotypes is associated with a disease phenotype, we need to test for whether *G*_*AA *_= *G*_*Aa *_= *G*_*aa *_or, equivalently, a null hypothesis of *H*_0_: *a *= *d *= 0 versus its alternative *H*_*a*_: *a *or *d *≠ 0. The standard regression approach can usually provide unbiased estimates of the model parameters and appropriate test for *H*_0 _regardless of possible correlation between w* (*g*), v* (*g*), although it may give large standard errors for the least square estimates of parameters when this correlation is very strong.

Now, let us look at the performance of model (5) on partition of genotypic variances. As w* and v* are two coding variables for the three genotypes at the same locus, they are inherently correlated. In fact, let *P*_*AA*_, *P*_*Aa*_, *P*_*aa *_be the genotype frequencies, we can show that Cov(w*, v*) = *P*_*Aa*_(*P*_*aa *_- *P*_*AA*_) ≠ 0 as long as *P*_*aa *_≠ *P*_*AA*_. They also have relationships with the index coding variables *w'*, *v' *in model (2) and the index variables *w*, *v *in model (3) as w* = *w' *- 1 = *w *+ 2*p *- 1, v* = *w' *- 2*v' *= (1 - *p*)*w *+ *v *+ (2*p *- *p*^2^).

Therefore, we have under HWE



In terms of the model parameters, we can show that *a *= *α *- (1 - 2*p*)*δ *and *d *= *δ*. In summary, we have the following conclusions.

• Model (5) usually provides a different partition of the genotypic variance *V*_*G *_than the one from the Cockerham model (3).

• When *P*_*aa *_= *P*_*AA*_, model (5) can give an orthogonal partition of the genotypic variance *V*_*G *_= *V *(aw*) + *V *(dv*), which is different from *V*_*G *_= *V*_*A *_+ *V*_*D *_in the Cockerham model (3) under the assumption of HWE unless .

• The potential correlation between w* and v* often leads to an increased share of *V *(dv*) other than *V*_*D*_, which is partly contributed by a portion of the additive variance.

• The dominance effect *d *is the same as the allelic interaction *δ *in the Cockerham model. As a result, *V*_*D *_= 0 if *d *= 0.

• The additive effect *a *= 0 is equivalent to *α *= (1 - 2*p*)*δ *for the allelic effects in the Cockerham model. So, a = 0 does not necessarily imply *V*_*A *_= 0.

Note also that making mean-corrections on the two coding variables w* and v* of genotypes does not help to separate their confounding in this case because dv* in model (5) is not an interaction term.

Extension of the F_∞ _model (5) to multiple QTL is straightforward. Still consider two loci **A **and **B **with alleles *A*, *a *and *B*, *b*, respectively. We can introduce variables  (*g*),  (*g*), i = 1,2, using the same '1 - 0 - (-1)' and '0 - 1 - 0' coding for QTL genotypes at each locus. Then a two-locus F_∞ _model with epistasis included yields

(6)

Model (6) is also a fully parameterized model for the 9 genotypic values *G*_*AB*_. As shown in Zeng *et al*. [7], this two-locus F_∞ _model can be written in a matrix form as , where  = (*m*, *a*_1_, *d*_1_, *a*_2_, *d*_2_, *aa*, *ad*, *da*, *dd*)^*T*^, and



When we fit the above model under a regression model framework, the expected mean of the least square estimates (LSE)  of  will be given by



where *W*_*AB *_= *diag*(*P*_22_, *P*_21_, *P*_20_, *P*_12_, *P*_11_, *P*_10_, *P*_02_, *P*_01_, *P*_00_) is of full rank with *P*_*ij *_being the frequency of genotypes corresponding to *G*_*ij*_, *i*, *j *= 0, 1, 2. So, the LSE provide unbiased estimates of , regardless of whether there are Hardy-Weinberg or linkage disequilibria in the genotypic distribution *P*_*g*_. However, as pointed out in Zeng *et al*. [7], the additive effect *a*_1 _can no longer be interpreted as a half of the difference between the homozygote genotypic values *G*_2 _= *E*(*G*|*AA*) and *G*_0 _= *E*(*G*|*aa*) at locus **A **in the presence of interaction effects, and so does the dominance effect *d*_1 _as the difference between the heterozygote genotypic value *G*_1 _= *E*(*G*|*aa*) and the mean of the homozygote genotypic values *G*_2_, *G*_0_. In addition, its partition of genotypic variance *V*_*G *_is complex because not only the within-locus terms *a*_*j *_ and *d*_*j *_ are correlated for *j *= 1, 2, but the within-locus terms {*a*_*j *_, *d*_*j *_} and the locus-by-locus interactions  could also be correlated. As a result, even when the genotypes at loci **A **and **B **are independent (i.e., the so-called zygotic equilibrium between loci **A **and **B **[18]), the variance component *V *(*a*_*j *_ + *d*_*j *_), *j *= 1, 2, cannot simply be interpreted as a variation contributed by locus *j *in the presence of interactions.

If we consider using the mean-corrected variables *ξ*_*j *_=  - *E *() and *η*_*j *_=  - *E *() to replace  and  for *j *= 1,2 in the F_∞ _model (6), this leads to the following model,

(7)

where



As in the one locus case, the mean-corrected variables *ξ*_*j *_and *η*_*j *_are very likely correlated within each locus *j *= 1, 2. But it could help to reduce the complexity of variance partition in certain circumstances. For example, under zygotic equilibrium between loci **A **and **B**, {*ξ*_1_, *η*_1_} are independent of {*ξ*_2_, *η*_2_}, and {*ξ*_*j*_, *η*_*j*_, *j *= 1, 2} are uncorrelated with interactions {*ξ*_1 _*ξ*_2_, *ξ*_1 _*η*_2_, *η*_1 _*ξ*_2_, *η*_1_*η*_2_} as well. As a result, the within locus effects (), j = 1,2, and the locus-by-locus interactions (*aa' ξ*_1 _*ξ*_2 _+ *ad' ξ*_1 _*η*_2 _+ *da' η*_1 _*ξ*_2 _+ *dd' η*_1 _*η*_2_) as a whole are orthogonal to each other, although the interaction terms {*aa' ξ*_1 _*ξ*_2_, *ad' ξ*_1 _*η*_2_, *da' η*_1 _*ξ*_2_, *dd' η*_1_*η*_2_} among themselves may still be correlated. Thus,



In general, for more than two loci under zygotic equilibria, we will have



In this case, *V *(*a*_*j *_ + *d*_*j *_) is the variation contributed by genotypes locus *j*, while *V *() represents the variation contributed by genotypic interactions between loci *j *and *k*. We will refer to model (7) as a mean-corrected F_∞ _model. It is interesting to see that, in an F_2 _population, this mean-corrected F_∞ _model is reduced to the classical F_2 _model as its special case. The same situation happens for the Cockerham model (4) as well.

We can also model multiple QTL by extending model (2) to multiple loci. For example, an allele-based two-locus biallelic model is given by

(8)

where ,  are coding variables defined in the same way as the ones in model (2) for the two loci separately. It is a model similar to the F_∞ _model (6) except that the coding variables of genotypes are defined in different ways. From the definition of these coding variables, it is also easy to see that  and . We can show that the parameters in models (8) and (6) have the following relationship



Without locus-by-locus allelic interactions, we have *a*_*j *_=  and  for *j *= 1, 2. In the presence of locus-by-locus allelic interactions, *a*_*j *_= *d*_*j *_= 0 is not equivalent to . As alleles represents the more basic levels of genetic factors than genotypes, the allele-based models are inherently more general and can be utilized to examine specific allelic effects and their interactions. When phase information is available, we could also use separate indicator variables of alleles to specify the paternal and maternal origins of alleles, which could be very useful in situations where the paternal or maternal genes may have different allelic effects and their interactions are of interest (e.g., genetic imprinting). On the other hand, the coefficients in a F_∞ _model are more closely associated with homozygosity and heterozygosity at the loci [2].

In regard to the modeling schemes, we can see that a major difference between the F_∞ _and Cockerham models lies in whether we treat genotypes or alleles as levels of the locus factors. The traditional F_∞ _models treat genotypes as levels of the locus factors with genotypic effects at each locus and locus-by-locus genotypic interactions being of major interest. The Cockerham models are defined by treating alleles as levels of the locus factors with a focus on partition of genotypic variances into various genetic variance components, and by using a mean-correction on coding variables of alleles it can effectively reduce the confounding between allelic effects and their interactions in partition of the genotypic variance. Both types of models can actually have two different versions - one is defined directly on coding of genotypes (or allele types), and the other on using mean-corrected index variables to reduce confounding between the main effects and their interactions. The former ones, either genotype-based or allele-based, have their coding variables defined on genotypes or alleles directly regardless of the genotypic or allelic distributions. The latter ones are based on some mean-corrected index variables, which depend not only on the genotypes or allele types but also on frequencies of these genotypes or alleles. To distinguish model parameters in these different models and meanwhile stay consistent with current terminology, in the rest of this paper we will simply refer to the additive, dominance and epistatic effects  in a traditional F_∞ _model as the *genotypic effects*; the parameters in a mean-corrected F_∞ _model as the *average genotypic effects *with their corresponding variance components as *genotypic variance components*; the parameters in an allele-based model (e.g., model (2) or (8)) which is defined based on the coding variables of allele types as the *allelic effects*; and parameters in the traditional (mean-corrected) Cockerham model as the *average allelic effects *with their corresponding variance components as *allelic variance components*.

Models directly using coding variables of genotypes or allele types are appealing in practice due to their simplicity. However, statistical tests of the genotypic or allelic effects based on p-values are highly dependent on the regression model, the distribution assumptions and the available sample size. A statistically significant genetic effect with a small p-value does not necessarily imply a clinically important finding. Besides, there could be inconsistency in definition of model parameters based on a one-locus model or a two-locus model with epistasis [7]. That is, when a multi-locus model is applied with epistasis involved, the interpretation of the additive and dominance effects based on one QTL model may change. On the other hand, using models with the mean-corrected index variables can allow us to assess how much variations are actually contributed by certain genetic effects or interactions, which could provide consequential information for achieving the clinical importance. A drawback in using these mean-corrected models is that they bring genotype or allele frequencies into the design matrix for regression, which will contribute another source of variation in fitting the model as the genotype or allele frequencies need to be estimated in practice. This fact could make it difficult to evaluate variance in estimates of the variance components.

The traditional (mean-corrected) Cockerham model can provide orthogonal partition of genotypic variance into additive, dominance and epistatic variance components under HWE and linkage equilibrium, while under zygotic equilibrium the mean-corrected F_∞ _model can give orthogonal partition of genotypic variances between different loci and locus-by-locus interactions. Which of the two mean-corrected models can provide simpler structure in partition of the genotypic variance really depends on the equilibrium situation in our sample. It is easy to see that a linkage equilibrium between alleles at two QTL under HWE can guarantee zygotic equilibrium of genotypes at the two loci but not the vice versa. Thus, for multiple QTL under both linkage and Hardy-Weinberg equilibria, the Cockerham model is preferred. When there is zygotic equilibrium of genotypes between two loci but no linkage equilibrium, a mean-corrected F_∞ _model might be preferred. In general, no one model is always preferable to the other in partition of genotypic variances. However, as HWE is expected to (or approximately) held in most of the human genomic regions, QTL with zygotic equilibrium but no linkage equilibrium are possible but rare. In addition, the allelic variance components are important quantities in assessing covariance between relatives and more closely related to the inheritance properties of quantitative traits. As a result, the allelic variance components based on the Cockerham model would expected to be of the main research interest in most of the cases for the genetic variance components analysis.

### Genotypic effects and allelic variance components

In Zeng *et al*. [7], it was pointed out that the additive, dominance and epistatic effects in an F_∞ _model and the average allelic effects in a Cockerham model are simply two different ways of re-parameterization for the genotypic values. They are transferable from each other through their relationship with the genotypic values when fully parameterized models are applied. Since partition of genetic variance components based on Cockerham models has been well established [14,21,22], a relationship between the genotypic effects in an F_∞ _model and the average allelic effects in its corresponding Cockerham model would allow us to compute various allelic variance components in terms of genotypic effects by translating those formulas on partition of genotypic variance derived from the Cockerham models based on the average allelic effects. In this section, we present detailed formulas for computing the allelic variance components in terms of the genotypic effects for the one-locus F_∞ _model (5) under Hardy-Weinberg disequilibrium and the two-locus F_∞ _model (6) with both epistasis and LD between the two loci. We also propose an alternative way of linking these two sets of parameters through the relationship between the coding variables of genotypes used in F_∞ _models and the mean-corrected index variables used in the Cockerham models. Some practical issues relating to using of reduced models instead of the fully parameterized models are also addressed.

Let us start from the simple case of the single locus F_∞ _model (5) and its equivalent Cockerham model (3). As pointed out in [7], we can build the relationship between the two sets of model parameters through the genotypic values. Since both models give a full parameterization of the three genotypic values *G*_*AA*_, *G*_*Aa *_and *G*_*aa*_, based on the coding functions for the three genotypes, we have



With some simply algebra, we can show that the genotypic effects and the average allelic effects have the following relationship



where *α *is the same substitution effect of replacing allele *a *by *A *as presented in [4] (p.114). Replacing *α*, *δ *in the formula (4) by *a*, *d*, we obtain the following partition of *V*_*G *_in terms of *a*, *d *in model (5)



Under HWE, we have *f *= 0. Then *V*_*A *_= 2*pq *[*a *+ *d*(*q *- *p*)]^2 ^and *VD *= 2(*pqd*)^2^. This is the same results that were presented in [4,20].

Similarly, for a two-QTL model (6), its genotypic effects  = (*m*, *a*_1_, *d*_1_, *a*_2_, *d*_2_, *aa*, *ad*, *da*, *dd*) and the average allelic effects *E*_*G*2*A*·*AB *_in its equivalent Cockerham model have the relationship , which yields

(9)

Assuming HWE at loci **A **and **B **but allowing LD between the two loci, by applying the properties of moment functions we derived before, it can be shown that the variance and covariance components in terms of average allelic effects in the two-locus Cockerham model (4) are given below



where *A*_1 _= *α*_1 _*w*_1_, *D*_1 _= *δ*_1 _*v*_1_, *A*_2 _= *α*_2 _*w*_2_, *D*_2 _= *δ*_1 _*v*_2_, *A*_1 _*A*_2 _= (*αα*)*w*_1 _*w*_2_, *A*_1 _*D*_2 _= (*αδ*)*w*_1 _*v*_2_, *D*_2 _*A*_1 _= (*δα*)*v*_1 _*w*_2 _and *D*_1 _*D*_2 _= (*δδ*)*v*_1 _*v*_2_. Note that the covariance components are caused by correlation between various allelic effects and interactions, while the interactions contribute their own variances regardless of whether the alleles are in HWE and LD or not. The above results are similar to what we presented in [14] for a general G2A model except that a more detailed partition of variance components and their covariance structures are shown here. Note also that the scales for defining the index variables *v*_1_, *v*_2 _here are slightly different by (-2) folds from the ones used in [14] to keep consistent with the ones used in Zeng *et al*. [7]. Correspondingly, those coefficients related to *v'*s in model (4) differ from the ones in [14] by (-2) or 4 folds depending on how many *v'*s are involved. Replacing the allelic effects in the above formulas by genotypic effects using their relationship (9), we can then obtain formulas of the variance and covariance components in terms of the genotypic effects for partition of the genotypic variance. When there is linkage equilibrium between loci **A **and **B**, then *D *= 0 and we have exactly the same result as presented in Tiwari and Elston [19].

In genetic applications, using fully parameterized models may not always be practical due to limited sample sizes, multiple QTL, or a large number of alleles or genotypes showing up at certain QTL. Including all possible genotypic or allelic interactions could make the genetic model over parameterized and hard to fit with too many parameters involved. Collapsing certain number of alleles or genotypes may simplify the model structure but dosing so could meanwhile increase the risk of losing detection of certain informative signals, as effects of true functional alleles can be attenuated by other non-functional alleles. By contrast, a simplified genetic model could be used to include only lower-order terms such as additive, dominance and additive by additive interactions.

Consider a simplified model from the previous two-locus F_∞ _model with only additive effects at the two loci and the additive by additive interaction being involved. Then, the reduced model is given by

(10)

In this case, we have



A reduced model can be thought of as adding constraints on the genotypic values. From , we now have



and *δ*_1 _= *δ*_2 _= (*α*_1 _*δ*_2_) = (*δ*_1 _*α*_2_) = (*δ*_1 _*δ*_2_) = 0. Thus, when there is HWE at loci **A**, **B **and linkage equilibrium between loci **A **and **B**, the partition of genotypic variance is given by , with



and .

If there is HWE at loci **A**, **B **but LD between the two loci, we will still have the same ,  and . Besides,



So far, we have relied on the equation  to establish the relationship between the average allelic effects *E*_*G*2*A*·*AB *_and the genotypic effects . Alternatively, we can establish the relationship between *E*_*G*2*A*·*AB *_and  through the coding variables used in the F_∞ _models and the index variables used in the Cockerham models. It is easy to see that the index variables ,  in the F_∞ _model (6) and *w*_1_, *w*_2 _in the Cockerham model (4) have the following relationship

(11)

for *i *= 1, 2. So, replacing w*, v* in model (10) by *w*, *v *gives



which leads to the the same results as we showed before. If there are dominance effects involved in the reduced model, then



It is easy to show that the relationship between the allelic effects *β *and the genotypic effects *b *is given by



Therefore, with the relationships (11), we can easily transform a F_∞ _model to its equivalent Cockerham model, or vise versa.

It must be pointed out that the above relationship between the genotypic effects and the average allelic effects hold only when the reduced F_∞ _models specify the genotypic values correctly. In practice, the true genotypic values are unknown and a reduced model can only provide an approximation of the true genotypic values. In this case, the least square estimates  from fitting a reduced model simply gives an unbiased estimator of the partial regression coefficients with expected mean



where *W*_*AB *_= *diag*(*P*_22_, *P*_21_, *P*_20_, *P*_12_, *P*_11_, *P*_10_, *P*_02_, *P*_01_, *P*_00_) is the same as defined before, ()^*g *^denotes a generalized inverse of the matrix (). In this case, the true parameters  may depend on not only the genotypic values but also the genotypic frequencies *P*_*g *_with possible allelic association such as LD involved - a fundamental difference between the statistical models and functional models as claimed in [17]. Furthermore, from the relationship , we can see that in general only certain linear combinations of *E*_*G*2*A*·*AB *_can be estimated from  because  may no longer be a non-singular square matrix. Thus, in this situation, some allelic variance components may not be directly estimable in terms of the genotypic effects in a reduced F_∞ _model. Alternatively, we can start from a reduced Cockerham model and derive its corresponding reduced F_∞ _model through using the relationship (11) when some allelic variance components can be reasonably ignored.

## Discussion

Nowadays, the F_∞ _models have been widely used in genetic association studies to test for genotypic association and their interactions with quantitative traits. Most of current association studies, however, focus on reporting p-values from statistical association tests on the additive, dominance and epistatic effects of QTL. As we have pointed out, an assessment of genetic variations contributed by these genetic effects to the overall genotypic variance is another important piece of information which could be consequential for achieving the clinical significance. Unfortunately, the calculation of either the genotypic or allelic variance components for F_∞ _models is not trivial, especially when QTL interactions and LD are involved.

In this paper, we first explored the modeling schemes for the F_∞ _and Cockerham models. We showed that the F_∞ _models are basically genotype-based models by treating genotypes as different factor levels, while the Cockerham models are allele-based models with a special modification to correct a collinearity problem. These two models usually provide different partitions of genotypic variances. Due to an inherent correlation between the additive and dominance effects within a locus in F_∞ _models, variances contributed by the within-locus additive and dominance effects are quite often confounded with each other. Therefore, separate assessment of variations contributed by the additive and dominance effects within a locus is not very meaningful. In order to fully capture the genotypic contribution at a locus, variations contributed by both additive and dominance effects should be assessed jointly.

In this paper, we also pointed that either the Cockerham or the F_∞ _model can have two different versions - one is defined based on some coding variables for allele types or QTL genotypes, and the other uses some mean-corrected index variables. Using those mean-corrected index variables can help to reduce the complexity in partition of genotypic variances under either linkage or zygotic equilibria. For example, the traditional (mean-corrected) Cockerham model can provide orthogonal partition of genotypic variance into additive, dominance and epistatic variance components under HWE and linkage equilibrium, while under zygotic equilibrium a mean-corrected F_∞ _model can provide orthogonal partition of genotypic variances between different loci and locus-by-locus interactions. By introducing the mean-corrected index variables, we can easily fit a Cockerham model or a mean-corrected F_∞ _model and compute various allelic or genotypic variance and covariance components using the standard regression approach. It was also noticed that the classical F_2 _model used in experimental designed populations is actually a special case of both the traditional (mean-corrected) Cockerham model and the mean-corrected F_∞ _model.

Using the mean-correction to dissect the confounding of main effects and their interactions on partition of variances is a useful strategy that can also be applied to evaluate gene by environmental interactions. Back to the original model (1), similar to the allelic effects and their interactions in model (2), the genetic main effect *G *and the gene by environmental interactions *G *× *E *could be correlated as well. This correlation usually does not affect the association test of the gene by environmental interactions. But it can lead to a covariance between the genetic main effect *G *and the gene by environmental interactions *G *× *E *on partition of the phenotypic variances even when the main effects of *G *and *E *are uncorrelated, which complicates evaluation of the variation contributed by *G *× *E*. If we make mean-corrections on both *G *and *E*, then we can obtain an orthogonal partition of the phenotypic variance *V*_*Y *_contributed by *G*, *E *and their interactions *G *× *E *as long as *G *and *E *are uncorrelated. Without making these mean-corrections, the variance *V *(*G *× *E*) itself could be an incorrect estimate of the actual variation contributed by *G *× *E *in addition to the genetic and environmental variances *V *(*G*) and *V *(*E*).

As allele-based models, we can easily incorporate some allele related properties such as HWE or linkage equilibrium into the variance components analysis for the Cockerham models. In this paper, we further explored some useful properties of the index variables and derived formulas of several important moment functions for a G2A model under LD. Similar results can be derived for more than two loci. With three loci **A**, **B **and **C**, for example, we can show that



for any integers *n*_1_, *n*_2_, *n*_3 _> 0. For more than three loci with linkage disequilibria, the moment functions will become more complex. But it is still computationally feasible as long as we have information about the haplotype distribution in the sampled population.

When there is no need to distinguish the paternal and maternal gametes, the F_∞ _and Cockerham models are transferable. There are two different ways of linking the genotypic effect parameters in a F_∞ _model with the allelic effect parameters in its corresponding Cockerham model - through either their relationship with the genotypic values, or the relationship between the coding variables of genotypes used in the two types of models. By establishing the relationship between the genotypic effects and allelic effects, we can then calculate the allelic variance components for a F_∞ _model using the partition formulas derived for its Cockerham model. Using this approach, for a one-locus F_∞ _model under Hardy-Weinberg disequilibrium and a two-locus G2A model with epistasis and LD, we presented detailed formulas for partition of genetic variances in terms of their genotypic effects. Moreover, some practical issues related to using of reduced instead of fully parameterized F_∞ _models were also addressed.

Both the F_∞ _and the Cockerham models are statistical models, as their model parameters depend on not only genotypic values but also the genotypic distribution in the sampled population especially when reduced forms of the models are used. On the other hand, several attempts have been made to model QTL effects and epistasis based on their biological functions. Cheverud and Routman [5] and Cheverud [6] introduced an unweighted F_∞ _model and defined several specific epistases termed as "physiological epistases". Hansen and Wagner [17] further inspected genotype-based interactions and termed them as "functional epistases". In our opinion, these two kinds of models are different ways of modeling genetic effects and their interactions, and they serve for different research interests. The function-based models could be very useful in analysis of the molecular functions of genes and their pathways. In genetic mapping studies, however, they are hindered by lack of appropriate model building tools and the fact that the genotypic values are statistically defined as expected means over the genotypic distribution in the sampled population. With a great body of available regression tools, the statistical models provide a powerful tool for detecting at least relatively common genes with certain magnitude of genetic effects in accommodation with the limited sample sizes.

## Conclusion

The Cockerham type models are allele-based models whereas F_∞ _regression models are genotype-based models. When allelic effects and their interactions are of main research interests, the Cockerham type models are recommended. As genotype-based models, the F_∞ _models are most suitable for examining genotypic effects and their interactions. Since the allelic variance components are important quantities in assessing covariance between relatives, the calculation and statistical tests of the allelic variance components would be helpful for assessing how much variations are actually contributed by the allelic effects at each locus, and the locus-by-locus allelic interactions, which could become a crucial piece of information for assessing the clinical importance. For a Cockerham model with the mean-corrected index variables, the allelic variance components can be estimated directly using the standard regression approach. For an F_∞ _model, when there is no need to distinguish the paternal and maternal allelic effects, we can transform it into its corresponding Cockerham model through the relationship between their coding variables of genotypes. Allelic genetic variance components for the F_∞ _model can then be calculated by either fitting its equivalent Cockerham model or translating formulas derived from the Cockerham model in terms of the additive, dominance and epistatic genetic effects in the F_∞ _model. Both the F_∞ _and Cockerham models provide basis for the QTL analysis. We believe that a better understanding of the differences and links between these two types of models will be helpful for genetic association mapping studies, variance components analysis and dissection of the genetic architecture of quantitative traits.

## Authors' contributions

TW participated in the design of the study, conducted the derivation and drafted the manuscript. ZBZ participated in the design of the study and edited the manuscript. All authors read and approved the final manuscript.
